# Correlative electrochemical strain and scanning electron microscopy for local characterization of the solid state electrolyte Li_1.3_Al_0.3_Ti_1.7_(PO_4_)_3_

**DOI:** 10.3762/bjnano.9.148

**Published:** 2018-05-28

**Authors:** Nino Schön, Deniz Cihan Gunduz, Shicheng Yu, Hermann Tempel, Roland Schierholz, Florian Hausen

**Affiliations:** 1 Forschungszentrum Jülich, Institute of Energy and Climate Research, IEK-9, 52425 Jülich, Germany; 2 RWTH Aachen University, Institute of Physical Chemistry, 52074 Aachen, Germany; 3 Jülich-Aachen Research Alliance, section JARA-Energy, 52425 Jülich, Germany

**Keywords:** correlative microscopy, electrochemical strain microscopy (ESM), Li_1.3_Al_0.3_Ti_1.7_(PO_4_)_3_ (LATP), scanning electron microscopy (SEM), solid state electrolytes (SSE)

## Abstract

Correlative microscopy has been used to investigate the relationship between Li-ion conductivity and the microstructure of lithium aluminum titanium phosphate (Li_1.3_Al_0.3_Ti_1.7_(PO_4_)_3_, LATP) with high spatial resolution. A key to improvement of solid state electrolytes such as LATP is a better understanding of interfacial and ion transport properties on relevant length scales in the nanometer to micrometer range. Using common techniques, such as electrochemical impedance spectroscopy, only global information can be obtained. In this work, we employ multiple microscopy techniques to gain local chemical and structural information paired with local insights into the Li-ion conductivity based on electrochemical strain microscopy (ESM). Scanning electron microscopy (SEM) and energy-dispersive X-ray spectroscopy (EDX) have been applied at identical regions to identify microstructural components such as an AlPO_4_ secondary phase. We found significantly lower Li-ion mobility in the secondary phase areas as well as at grain boundaries. Additionally, various aspects of signal formation obtained from ESM for solid state electrolytes are discussed. We demonstrate that correlative microscopy is an adjuvant tool to gain local insights into interfacial properties of energy materials.

## Introduction

Solid state electrolytes (SSE) of the NASICON-type exhibit a high ionic conductivity and are in this respect becoming comparable to conventional organic electrolytes commonly used in lithium-ion batteries (LIBs) [1–5]. SSEs have gained much interest in recent years for replacing the flammable liquid electrolyte in LIBs, especially in safety-related environments like automotive applications [6–7]. Furthermore, the increased electrochemical window in the case of SSEs opens the path to use advanced electrode materials with improved volumetric and gravimetric energy density [1,8–10]. Lithium aluminum titanium phosphate Li_1.3_Al_0.3_Ti_1.7_(PO_4_)_3_ (LATP), a ceramic with NASICON-type structure, is especially considered as a beneficial solid state electrolyte due to its superior lithium-ion conductivity in the range of 2 mS cm^−1^ in the “bulk” and 2 µS cm^−1^ at grain boundaries with an overall conductivity of 0.2 mS cm^−1^ [11] and has therefore attracted much research within the last decade [12–15]. In classical electrochemical impedance spectroscopy (EIS), the ionic conductivity is measured through the entire sample and over the full electrode contact area (typically in the range of 1 cm^2^). Hence, only averaged values are obtained whilst locally the ion mobility can still be inhomogeneous [11]. Translating local ion migration into global conductivity is part of ongoing research. First approaches for small-scale impedance measurements have been reported to gain adequate EIS resolution by coupling with AFM [16]. The authors reported experiments on silver-ion conducting glasses and found good agreement between the mean value of local conductivities and the macroscopic conductivity. It has been found that the electrochemical characteristics of LATP correlate with the microstructure of the material [17–19]. The microstructure describes the relationship between density, porosity and particle size, grain structure and phase composition. These attributes are primarily defined by the sintering process [20] and have been analyzed macroscopically. But, as it can already be seen from the results of different lithium-ion conductivities for grain and grain boundary structures in comparison to the overall ionic conductivity, it is of utmost importance to understand the electrochemical and ion-transport properties of promising SSEs such as LATP at the length scale of the grain size, local defects and structural inhomogeneities, that is, on the nanometer to low-micrometer scale [10–11].

Reports on local behavior and properties of SSEs are scarce. Very recently, Sasano et al. have reported about the qualitative relation between grain orientation and Li-ion mobility in Li_0.33_La_0.56_TiO_3_ using scanning electron microscopy (SEM) with electron backscatter diffraction (EBSD) and electrochemical strain microscopy (ESM) [21]. The authors correlate variations in the Li-ion mobility detected by ESM with limitations in the Li-ion migration pathway. ESM is a relatively new technique based on atomic force microscopy (AFM): An AC voltage with the same frequency as the contact resonance frequency of the tip–sample contact is applied to a conductive tip. [22–23]. The induced electrical field in the material under investigation is extremely localized due to the small tip radius on the order of 15 nm. Hence, the interaction between the electric field and the local structure of the material can be studied with high spatial resolution. Mobile ions are accelerated by the electric field towards or away from the tip. Consequently, the concentration of ions changes within a small volume under the tip, leading to a deformation of the surface. The resulting strain is measured by the system and reflected in the ESM amplitude signal. For electrode materials, the strain is supposed to be directly proportional to the Li-ion mobility [24–27].

In this work we combine the strengths of two microscopy techniques: SEM in combination with energy-dispersive X-ray spectroscopy (EDX) and ESM as an AFM-based technique. Both techniques are consecutively employed at identical regions of interest on LATP samples. Hence, chemical information, as detected by EDX, and information about the local mobility of ions, extracted from ESM measurements, are available with very high spatial resolution. Such a correlative microscopy approach allows for direct comparison of microstructure and ionic mobility, enabling unique local insights into the structural, chemical and electrochemical characteristics of solid state electrolytes.

## Results and Discussion

**Figure 1:**
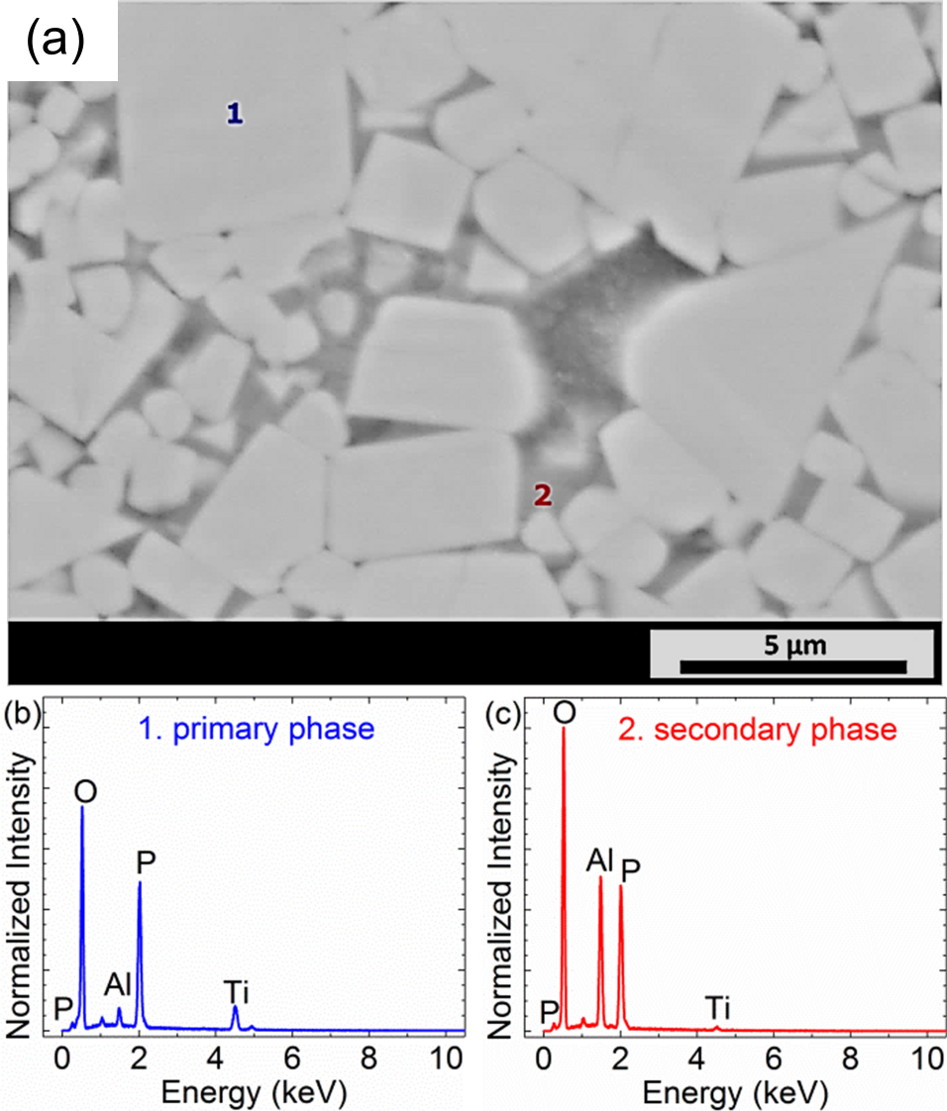
(a) Typical SEM back scattered electron (BSE) image of a LATP pellet sintered at 1000 °C and polished by hand; the markings 1 and 2 denote regions where EDX was performed. (b) Micro-area EDX spectra of a brighter grain of the material as denoted by the marking 1 in (a). The spectrum contains characteristic peaks corresponding to Al, Ti, O and P with intensities as expected for Li_1.3_Al_0.3_Ti_1.7_(PO_4_)_3_. (c) Micro-area EDX spectrum of a darker region of the material as denoted by the marking 2 in (a).

The grain structure of a typical region of hand-polished Li_1.3_Al_0.3_Ti_1.7_(PO_4_)_3_ (LATP), as observed by SEM, is shown in Figure 1a. A large variation in grain size and shape is clearly visible, with a preference for cubic structures. The largest grains are about 20 µm^2^ in size while the typical grain size is on the order of 1 µm^2^. As the surface was polished, the observed contrast in color cannot be related to topographical effects, but rather indicates the existence of a secondary phase. This finding becomes evident in EDX measurements, depicted in Figure 1b,c, revealing the existence of two separate phases inside the material. The primary phase (denoted as 1 in Figure 1a) appears brighter in the SEM image and consists of Al, Ti, P and O (Li is not detectable by EDX) while the secondary phase (denoted as 2 in Figure 1a) appears darker and contains the elements Al, P and O but only minor Ti content. Based on this observation, the primary phase is attributed to LATP while the secondary phase can be related to aluminum phosphate (AlPO_4_). As the stoichiometry of the EDX analysis matches that of LATP and aluminum phosphate, respectively, the assignment of the individual phases to their chemical composition is further supported. Additionally, the occurrence of a secondary phase of AlPO_4_ has been previously observed [1,4]. No changes based on the different composition in phase images of tapping-mode AFM, nor in peak-force tapping quantitative nanomechanical property mapping have been observed. The insignificant amount of detected titanium is due to the resolution limit of the EDX measurements. The volume from which characteristic X-ray peaks can escape is largest for Ti at Kα at 4510 eV, and therefore, contributions from the surrounding material should also be considered for Ti. For P (Kα at 2010 eV) and Al (Kα at 1486 eV) this volume becomes smaller and for O (Kα at 525 eV) only regions close to the surface contribute.

Recently, a very similar microstructure of LATP has been published and discussed in further detail [11,17]. Information about the behavior of the material as a solid state electrolyte cannot be derived based on SEM and EDX mappings alone, hence we performed ESM.

**Figure 2:**
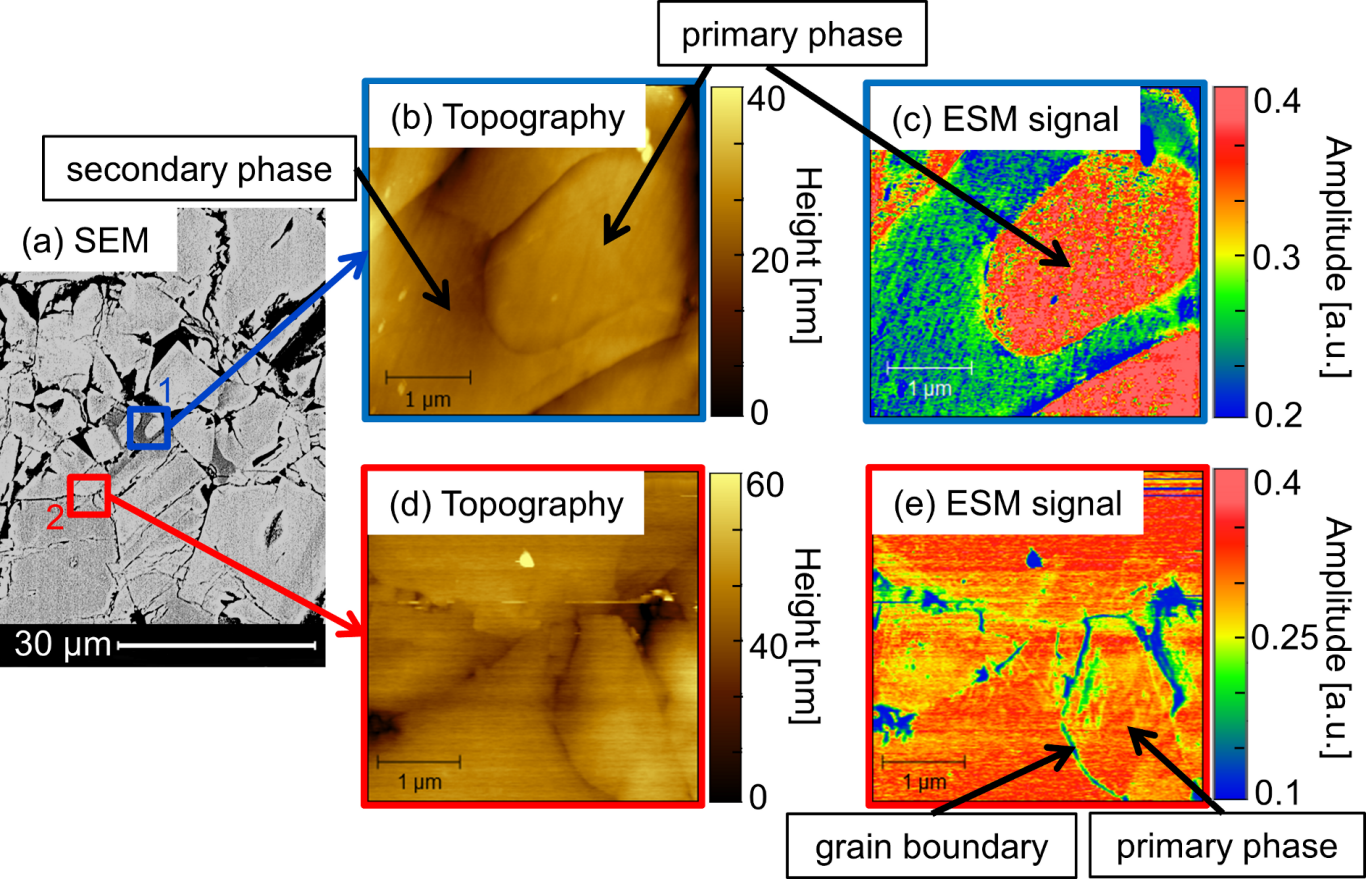
Correlative microscopy of selected areas on LATP sintered at 1050 °C and polished by hand. (a) SEM is used to identify grains of LATP and aluminum phosphate, respectively. (b) and (d) are topographical AFM images of the regions marked by blue and red squares in (a). (c) and (e) are ESM amplitude signals and exhibit a strong contrast between primary and secondary phase. The contact resonance frequency of the tip–sample system was at 281 kHz for (c) and 285 kHz for (e).

Figure 2 shows correlative images of SEM and AFM topography as well as ESM on identical regions of LATP sintered at 1050 °C. The SEM image (Figure 2a) illustrates the grain structure of the sample and reveals the existence of primary phase and secondary phase represented by different colors. In accordance with Figure 1 and the EDX spectra, such regions are attributed to LATP (brighter contrasts) and aluminum phosphate (darker contrasts), respectively. Additionally, the presence of several small pores is very likely as can be seen by the dark regions in Figure 2a, but these are mainly excluded for the regions selected for ESM measurements (except the upper-right corner in Figure 2b and the pore in Figure 2d). Correlative AFM and ESM images were obtained from two different areas indicated by the blue (1) and red (2) markings in Figure 2a. In the topography images illustrated in Figure 2b,d, minor amounts of residue originating from the polishing procedure are observed in the form of elevated particles. Apart from this, the AFM images reflect the same surface features as observed by SEM, providing evidence that both methods can be applied complementary. The same pores as those observed via SEM can be found in the AFM topography image and the topography reveals some preferential etching at the grain boundaries and interfaces. Differentiation between the primary and secondary phase is not possible based on the topography images as no isolated terraces with differing heights are formed.

As small pores often resemble a similar color as the secondary phase in SEM images, and are therefore difficult to distinguish from one another, correlative microscopy offers the advantage to unambiguously verify small-sized pores due to the high *z*-contrast of the AFM, as depicted in Figure 2d.

Next to the topography, the ESM amplitude signal for the regions indicated by blue and red squares in Figure 2a is recorded and depicted in Figure 2c,e. A change in the amplitude can be correlated to local interactions between the applied electrical field and the SSE material in the respective areas. While no differentiation between the primary and secondary phase of LATP could be derived from topography images, the simultaneously recorded ESM amplitude signal exhibits clear contrast between the different phases. According to the SEM image, it is possible to identify the grains as LATP and aluminum phosphate as labeled in Figure 2. It becomes apparent that LATP shows a strong ESM amplitude signal. In contrast, the amplitude is significantly smaller for regions consisting of aluminum phosphate. The residual particles on the top of the sample exhibit very low amplitude. In line with reports by Balke et al. and Sasano et al. [21–22,24] we correlate a larger ESM amplitude signal to higher Li content or Li mobility. A thorough discussion about signal formation follows later in the manuscript.

The selected regions of interest have been chosen as in area 1 (blue square) where grains of both LATP and aluminum phosphate are present, and hence, different Li-ion transport properties are to be expected. This assumption is verified by the larger ESM amplitude signal for the primary phase in comparison to the secondary phase, as demonstrated in Figure 2c. In spot 2 (red square), only LATP is present, but well-separated into several grains. A similar finding as for area 1 is obtained for the amplitude from spot 2 as demonstrated in Figure 2e. A strong amplitude signal was found for grains consisting of LATP. Interestingly, the grain boundaries exhibit significantly smaller amplitudes comparable to that of aluminum phosphate in area 1.

**Figure 3:**
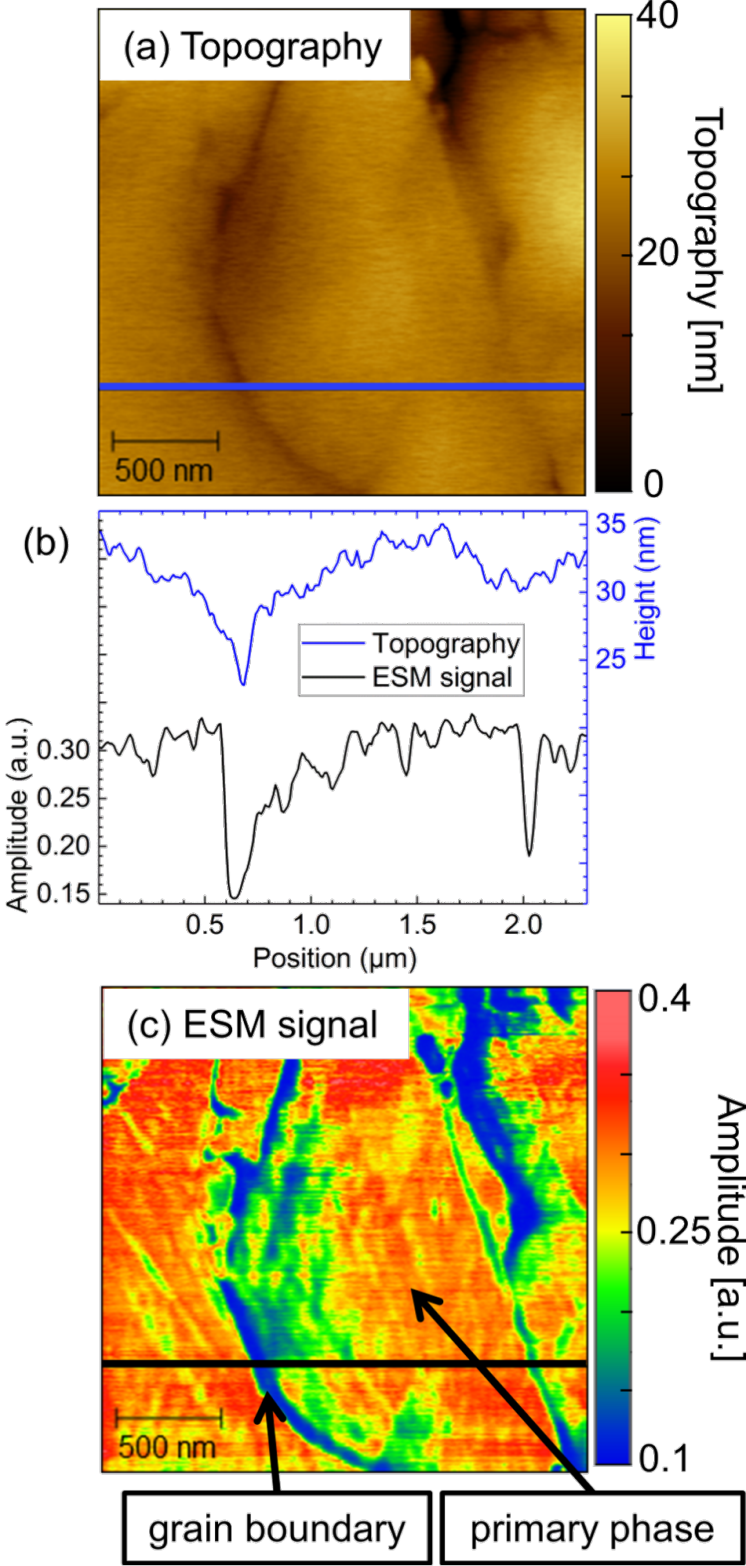
Higher resolution images of part of the area shown in Figure 2d and Figure 2e. (a) Topography, (c) ESM amplitude, and (b) line scans along the topography (blue) and the ESM amplitude signal (black) as indicated in the corresponding images. The contact resonance frequency of the tip–sample system was 299 kHz.

This is examined in more detail for the region of grain boundary marked by the arrow in Figure 2e. Figure 3 shows AFM topography and ESM images with higher resolution. Various effects are observed in the ESM amplitude signal, depicted in Figure 3c: Firstly, minor variations in the magnitude of the ESM amplitude signal are recognizable in Figure 3c in comparison to Figure 2e on the left side of the central grain. The image in Figure 3c was recorded about twenty minutes after Figure 2e. Such alterations demonstrate the dynamic nature of the system. Secondly, it is apparent from Figure 3c that the ESM amplitude signal shows differentiations inside individual grains of the primary phase. The origin of the effect of variations in the observed ESM amplitude intensity might be related to deviations in the lithium content within single grains. Besides, variations in crystal orientation might be accountable for the observed effect, as was discussed recently for LLTO [21]. However, both assumptions need further experimental evidence and cannot be answered based on the present data.

Finally, Figure 3c demonstrates that the grain boundaries between three grains of primary phase exhibit significantly smaller ESM amplitude signal than the grains themselves. The influence of the topography can be elucidated by the line sections as indicated in Figure 3a for topography and in Figure 3c for the ESM amplitude signal and shown in Figure 3b. From left to right three grains and correspondingly two grain boundaries can be identified in the topography image (Figure 3a). An overall height difference of about 8 nm for the left grain boundary and of 3 nm for the right grain boundary can be extracted from the line section illustrated in Figure 3b. For both grain boundaries, sharp peaks with significantly lowered magnitude of the ESM amplitude signal with respect to the overall grain are obtained at the point of lowest topography, i.e. the grain boundary. From the line section, it becomes clear that this observation cannot be related to an image or tip artifact as the simultaneously recorded topography exhibits a different and well-resolved response. While the ESM signal amplitude stays rather homogeneous across the grains, the topography shows a smoother transition from one grain to the other over a distance of about 0.7 µm to 0.8 µm. This finding indicates low crosstalk between the topography of the sample and the ESM amplitude signal. However, it is conceivable that the surface topography influences the observed ESM amplitude signal in some way. Topographical features lead to a change in contact area between tip and sample, which is likely to influence the signal formation process and therefore induce crosstalk as a change in contact area influences the contact resonance conditions. In order to investigate this effect in more detail, a different preparation method has been employed to obtain a significantly smoother LATP surface.

**Figure 4:**
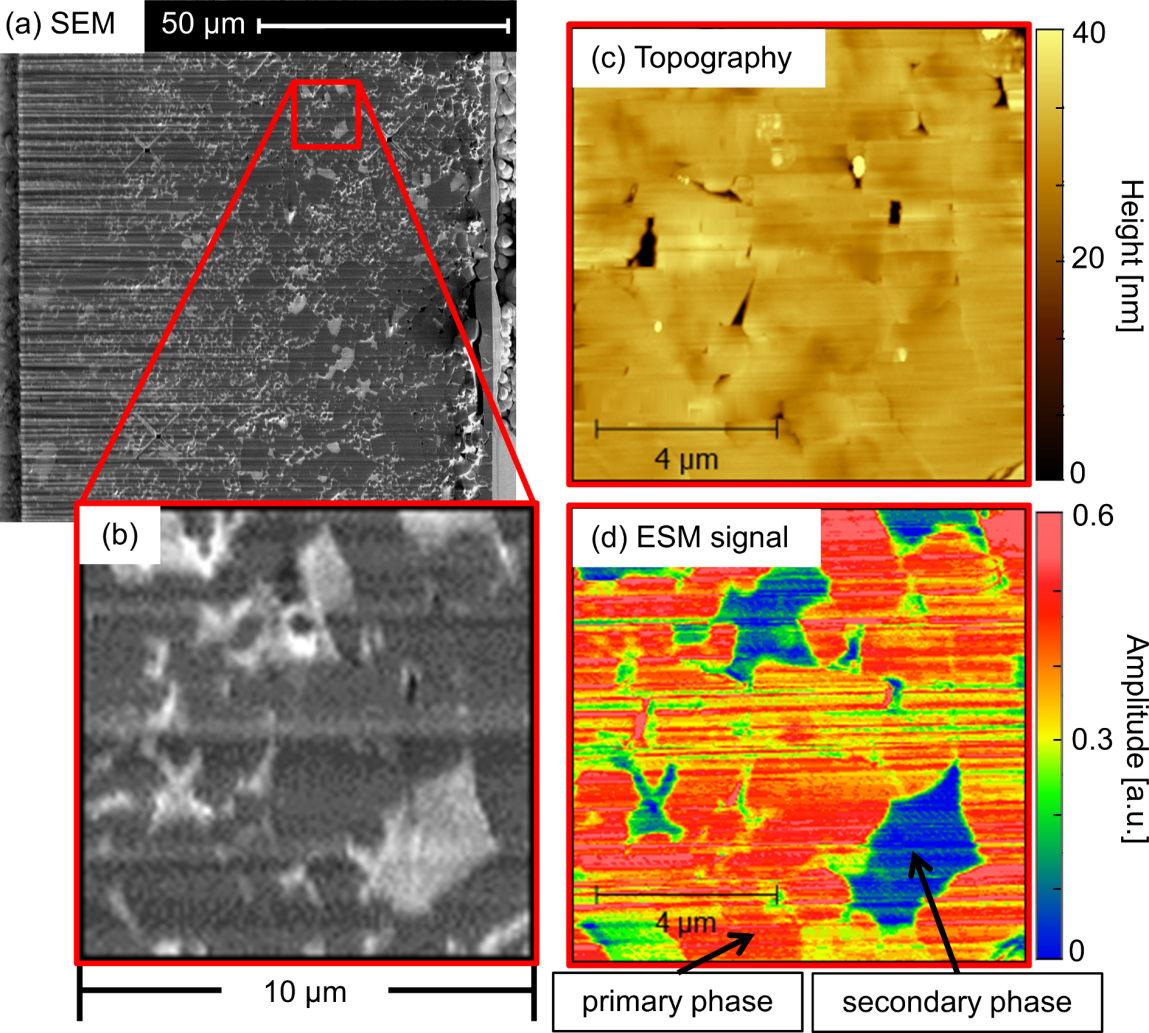
Correlative microscopy images from LATP sintered at 1000 °C and polished by a focused-ion beam. (a) SEM image of the polished area on the LATP pellet. In the left part of the image, a curtaining effect is observed. (b) Cut-out area of interest of 10 × 10 µm indicated by the red square revealing primary and secondary phases. (c) Topography and (d) ESM amplitude signal of the selected area shown in (b). The contact resonance frequency of the tips–sample system was 331 kHz.

Figure 4 shows an SEM picture of the area on LATP sintered at 1000 °C that was polished by means of a focused ion beam (FIB). Please note that the color contrast is inverted in this case as the SEM signal is detected by secondary electrons (SEM-SE) rather than back-scattered electrons (SEM-BSE). Therefore, darker regions correspond to LATP and brighter contrasts can be attributed to aluminum phosphate. Correlative microscopy was performed in a region of 10 µm × 10 µm in the selected area depicted in Figure 4b. AFM topography as well as ESM amplitude signal is shown in Figure 4c,d, respectively. In comparison with hand polished samples (see Figure 2b,d) a smoother surface finish was obtained. Nevertheless, the Ga-ion beam produced some trenches due to the curtaining effect. The hand-polished LATP sample exhibits an RMS roughness of 4.4 nm and 2.7 nm for the regions depicted in Figure 2b and Figure 2d, respectively. However, the sample shown in Figure 4c prepared by FIB exhibits an RMS roughness of only 1.8 nm for a comparable size.

Regardless of the curtaining effect, grain boundaries and individual grains can hardly be seen in the topography image (Figure 4c), using the same color scale as in Figure 2. This means, preparation with the ion beam almost parallel to the surface introduces less preferential etching of the grain boundaries. However, the ESM amplitude signal, as shown in Figure 4d, possesses a stark contrast between the primary and secondary phase. A considerably larger signal is observed for the primary phase that corresponds to LATP in comparison with aluminum phosphate, the secondary phase. The variation of the AFM scanning angle affected the observed lines, providing evidence that the effect results from a physical deformation of the sample surface induced by the FIB preparation.

As expected, grain boundaries display an ESM amplitude signal that is larger than for the secondary phase but smaller than for the primary phase. Significantly more “noise” is observed in Figure 4d and seems to appear in horizontal lines, which was the direction of the ion beam during preparation and therefore is introduced by the curtaining effect. However, at similar positions in the SEM image (Figure 4b), the curtaining effect is visible, which is a consequence of the FIB polishing and caused by local inhomogeneities in the material, leading to uneven erosion. This points to an influence due to the preparation method on the obtained ESM amplitude signal and should be considered for further experiments.

**Figure 5:**
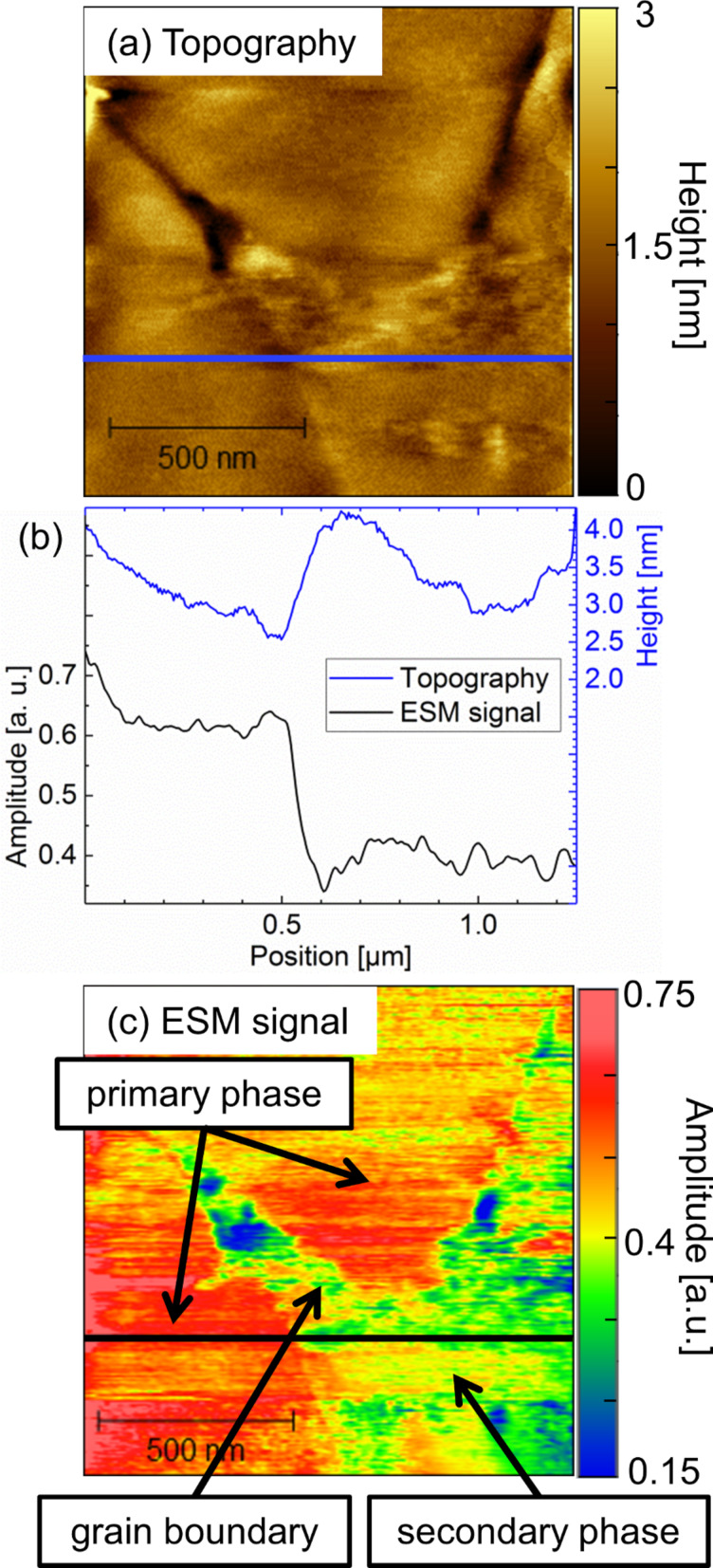
High magnification image of LATP polished by focused-ion beam showing the (a) topography and (c) ESM amplitude signal of the primary phase (LATP), secondary phase (AlPO_4_) and grain boundaries. (b) Line scans (average of ten lines) along the topography (blue) and the ESM amplitude signal (black) as indicated in the corresponding images. The contact resonance frequency of the tip–sample system was 292 kHz.

Topography as well as variations in the ESM amplitude signal with respect to grain boundary, primary and secondary phase on a higher magnification region for the FIB-polished sample are demonstrated in Figure 5. As observed before, a difference between the primary and secondary phase cannot be deduced by only topographical data from AFM. On the other hand, the ESM amplitude signal shows a pronounced contrast between different phases, whereas grain boundaries and the secondary phase are hardly distinguishable. This observation can be explained by a partial incorporation of AlPO_4_ into the grain boundaries. A similar effect has been recently reported as a function of sintering temperature, supporting the argumentation [17]. Of note is that at certain positions in the grain boundary a high ESM amplitude signal is also observed, suggesting a locally heterogeneous composition of the grain boundary. The negligible difference in ESM amplitude signal between the grain boundary and secondary phase also indicates what was previously demonstrated in Figure 2 and Figure 3, that is, significant contributions of the surface topography on the ESM amplitude signal are not likely to influence the results for the material studied here.

Assuming that a larger ESM amplitude signal corresponds directly to an increased Li-ion mobility in the sample, as predicted for Li-ion battery electrodes [22], the conclusion drawn in this manuscript is that Li-ion conductivity through the grains is favorable [11]. We want to emphasize that additional effects, such as a change in tip contact radius as well as changes in the crystal orientation, might also relate to the observed reduced ionic conductivity in grain boundaries. Correlative microscopy was successfully implemented to determining the influence of secondary phases on ion conductivity by enabling clear chemical analyses of each grain probed by ESM.

However, it is currently discussed to what extent the ESM amplitude signal can be attributed to an increased molar volume induced by the electric field in the vicinity of the tip, as proposed by Balke et al. [22]. This mechanism implies that the mobile ions in the material are attracted towards the surface, causing a strain of the material according to Vegard’s Law [25]. In the case of a solid state electrolyte, with its inherently low electronic conductivity, it remains arguable if the ESM amplitude signal is predominantly caused by ion migration. Very recently, Lushta et al. [28] presented ESM measurements on a commercially available Li-ion conducting glass ceramic (LICGC) using dual AC resonance tracking (DART) and band excitation (BE) as excitation methods. Furthermore, the authors calculated the diffusion constant and diffusion time of the commercially available LICGC, taking into account the density of the ceramic and the electronic conductivity. The idea behind this approach is that for reasons of charge neutrality, the mobility of cations is also linked to the electronic conductivity of the material. The authors came to the conclusion that the observed ESM signal cannot be based on Vegard’s strain, but rather on ion diffusion because the calculated diffusion processes are too slow to follow the applied AC frequency. As the calculations are independent of the exact excitation method we calculated diffusion coefficients for the LATP pellets used in this study according to equation 6 in [28] of 3.1 × 10^−16^ m² s^−1^ for pellets sintered at 1000 °C and of 3.0 × 10^−16^ m² s^−1^ for pellets sintered at 1050 °C. For the calculations a Li-ion conductivity of 2 mS cm^−1^, an electronic conductivity of 10^−10^ S cm^−1^ (as found in [29]), a temperature of 298 K as well as a density of 2.61 g cm^−3^ for pellets sintered at 1000 °C and 2.65 g cm^−3^ for pellets sintered at 1050 °C (both values taken from [16]), leading to a lithium ion density of 5.2 × 10^21^ cm^−3^ and 5.4 × 10^21^ cm^−3^, respectively, were considered. Assuming a diffusion length of 50 nm (as suggested in [28]) this leads to diffusion times of 8.1 s and 8.5 s, respectively. Even for a diffusion length of only 10 nm, a diffusion time of about 0.3 s is expected, which is far above the applied AC frequency on the order of 300 kHz. Based on these calculations, we also expect that effects other than Vegard’s Law contribute substantially to the observed ESM amplitude signal. Electrostatic interactions are discussed to be an important additional parameter that can influence ESM experiments [28,30–31] and will be the subject of future research. Additionally, LATP is known to have a strong anisotropic thermal expansion [3]. LTP, which has the same structure, exhibits an anisotropic reaction upon lithium intercalation; the *a*-axis contracts while the *c*-axis expands with the transformation from LiTi_2_(PO_4_)_3_ to Li_3_Ti_2_(PO_4_)_3_ [32]. Hence, LATP might show similar behavior upon (de)-lithiation and its electrostatic interaction can be expected to be strongly anisotropic. A simple interpretation of the ESM amplitude signal solely based on Vegard’s Law is therefore demanding and would require a correlative EBSD analysis of the area to retrieve the actual crystallographic orientation [21]. However, a thorough analysis is out of the scope of the current study but is part of ongoing research.

## Conclusion

We have presented correlative microscopy experiments by means of SEM and AFM-based techniques of the solid state electrolyte lithium aluminum titanium phosphate (LATP). In the SEM images, a primary and a secondary phase have been identified and could be attributed to LATP as the primary phase and AlPO_4_ as the secondary. ESM was employed to locally identify regions of increased interaction of the material with an applied alternating electric field. It was found that the secondary phase exhibits significantly lower interaction than the primary phase. It was discussed whether the interaction could be directly linked to Li-ion mobility. Furthermore, grain boundaries have been analyzed and show only weak response in ESM amplitude signal. This result is explained by incorporation of AlPO_4_ into the grain boundary structure, as suggested in the literature [11,17]. It has been proven that correlative microscopy leads to improved understanding of the microstructure–property relationship of solid state electrolytes on a process-relevant scale. Increased knowledge in this respect is of utmost importance in order to develop SSEs with better functionalities.

## Experimental

### Synthesis of Li_1.3_Al_0.3_Ti_1.7_(PO_4_)_3_ (LATP)

The synthesis of Li_1.3_Al_0.3_Ti_1.7_(PO_4_)_3_ (LATP) has been described in detail elsewhere [17] and consists of an oxalic acid supported sol–gel process. Binder-free dense LATP pellets as used in this study were obtained by an improved route involving preannealing, shaping, pressing and sintering in air at 1000 °C or 1050 °C for 8 h as indicated. For both AFM as well as SEM measurements, the pellets were subject to a polishing step by hand polishing or by focused-ion beam.

### Hand polishing

Smooth LATP surfaces suitable for SEM and AFM analysis were achieved by oil-based polishing to minimize exposure of LATP to water. For the first grinding step, silicon carbide paper of 800 grit was used with a particle size of about 20 µm. Step-wise, finer grit sandpapers were used: 1200 (15 µm), 2400 (10 µm) and ending with 4000 (5 µm). At each step, the sample was ground for 10 to 15 minutes at 150 rpm. This was followed by polishing the samples for 4 to 8 minutes at 300 rpm in four steps. In the first two steps, a diamond suspension with 3 µm and 2 µm particles was used. Finally, two finishing steps were performed with a 0.2 µm silica suspension and a 0.05 µm master polish. Afterwards, the pellets were rinsed thoroughly with isopropanol.

### Focused-ion beam polishing

For comparison, a second sample was additionally polished using a focused-ion beam (FEI (now Thermo-Fisher) Helios 460F1) [33]. For this, a polished SEM sample was used. A protective Pt-layer the size of the final polished surface (90 µm × 90 µm) was deposited by the ion beam. Around this protective layer, wedge-shaped trenches were cut and from the edge of the polished pellet material was removed with the cleaning cross-section pattern until the protective Pt-layer was reached. Then, the sample was rotated and tilted for perpendicular ion polishing. For this purpose again, a protective Pt-layer was deposited on the new cross-section before removing the initially deposited protective Pt-layer of 90 µm × 90 µm size as well as parts of the material beneath it, to form the ion-polished surface. Due to the large area, curtaining is present in the left part of the ion-polished area in Figure 4a. The protective Pt-layer and an incidence angle smaller than 1.5° precludes the material from being doped by gallium.

### Scanning electron microscopy

A SEM (Quanta FEG 650; FEI part of Thermo-Fischer, Hillsboro, Oregon, USA) equipped with a field emission gun (FEG) and EDX (Octane 60 mm^2^, EDAX Inc., Mahwah, NJ, USA) was employed to visualize the grain structure of LATP samples. Chemical information from the various regions of interest was obtained by means of EDX.

### Atomic force microscopy and electrochemical strain microscopy

An AFM (Bruker, Santa Barbara, USA, Dimension Icon Microscope) operating inside a glovebox (MBraun, Stratham, USA) was used to record AFM images. Electrochemical strain microscopy (ESM) is a special mode of the AFM, suitable to qualitatively detect local variations in ionic mobility [22]. As cantilevers, Bruker SCM-PIT-V2 (Bruker, Camarillo, USA) cantilevers with a conductive Pt/Ir coating and a nominal spring constant of 3 N·m^−1^ were employed. The contact resonance frequency and the amplitude were tracked with a phase-locked loop (HF2LI, Zurich Instruments, Switzerland) [34]. Further information on how to connect this instrument to a Bruker Dimension Icon AFM is described in [35]. The applied AC frequency must match the contact resonance frequency of the cantilever used, and is exactly given in the respective figure caption. To ensure a stable tip–sample interaction, a slow scanning speed of about 0.2 Hz was applied. Topographical images as well as the change in amplitude signal were recorded simultaneously. The samples were first investigated using SEM and subsequently using AFM.
